# Genetic Analysis of HIV-1 Subtypes in Nairobi, Kenya

**DOI:** 10.1371/journal.pone.0003191

**Published:** 2008-09-11

**Authors:** Suhail Khoja, Peter Ojwang, Saeed Khan, Nancy Okinda, Reena Harania, Syed Ali

**Affiliations:** 1 Department of Biological and Biomedical Sciences, Aga Khan University Hospital, Karachi, Pakistan; 2 Department of Pathology, Aga Khan University Hospital, Nairobi, Kenya; 3 Department of Medicine, Aga Khan University Hospital, Nairobi, Kenya; Institute of Human Virology, United States of America

## Abstract

**Background:**

Genetic analysis of a viral infection helps in following its spread in a given population, in tracking the routes of infection and, where applicable, in vaccine design. Additionally, sequence analysis of the viral genome provides information about patterns of genetic divergence that may have occurred during viral evolution.

**Objective:**

In this study we have analyzed the subtypes of Human Immunodeficiency Virus -1 (HIV-1) circulating in a diverse sample population of Nairobi, Kenya.

**Methodology:**

69 blood samples were collected from a diverse subject population attending the Aga Khan University Hospital in Nairobi, Kenya. Total DNA was extracted from peripheral blood mononuclear cells (PBMCs), and used in a Polymerase Chain Reaction (PCR) to amplify the HIV *gag* gene. The PCR amplimers were partially sequenced, and alignment and phylogenetic analysis of these sequences was performed using the Los Alamos HIV Database.

**Results:**

Blood samples from 69 HIV-1 infected subjects from varying ethnic backgrounds were analyzed. Sequence alignment and phylogenetic analysis showed 39 isolates to be subtype A, 13 subtype D, 7 subtype C, 3 subtype AD and CRF01_AE, 2 subtype G and 1 subtype AC and 1 AG. Deeper phylogenetic analysis revealed HIV subtype A sequences to be highly divergent as compared to subtypes D and C.

**Conclusion:**

Our analysis indicates that HIV-1 subtypes in the Nairobi province of Kenya are dominated by a genetically diverse clade A. Additionally, the prevalence of highly divergent, complex subtypes, intersubtypes, and the recombinant forms indicates viral mixing in Kenyan population, possibly as a result of dual infections.

## Introduction

Human Immunodeficiency Virus (HIV) has several perplexing attributes that distinguish it from other viruses. Important determinants of the pathogenicity of this virus are its genetic heterogeneity, which results mainly from the error-prone reverse-transcriptase activity during viral replication (introducing an average of one error per genome per replication cycle, the rapid turn over of HIV-1 *in vivo*, recombination (which occurs at a rate of about 2% per kilobase per replication cycle), and selective immune pressure by the host [1]–[5].

HIV has been primarily classified on the basis of geographical distribution and animal source of human infection into two types, HIV-1 and HIV-2. Both of these types can be transmitted through sexual contact and blood, as well as from mother to child, and are capable of causing AIDS. The propensity of the virus towards genetic diversity leads to rapid emergence of new types and subtypes that evolve further into subtypes, recombinant forms and quasispecies that are specific to infected groups and communities [6]–[11]. Exhibition of tremendous genetic variation in HIV-1 due to high mutation rates and recombination [12] has led to classification of the virus into three distantly related groups; Main group (M), Outlier group (O) and non-M-non-O group (N). The M group that dominates the AIDS pandemic has been subdivided into at least 12 distinct lineages, designated as subtypes and sub-subtypes (A1, A2, B, C, D, F1, F2, G, H, J, K and L) and almost 33 circulating recombinant forms (CRF) [13]–[15]. Viral epidemiological studies therefore provide opportunities to monitor the global spread of HIV, tracking routes of infection and analyzing patterns of virus' genetic divergence [9].

Earlier studies have demonstrated that HIV-1 subtypes are not randomly distributed among the globe and show distinct geographical distribution [16], [17]: Subtypes A and D are predominant in Africa; subtype B in USA, Europe, Australia, Thailand and Brazil; subtype C in South Africa, Ethiopia and India; F in some regions of Central Africa and Eastern Europe and CRF01_AE in southeast Asia [4], [18]–[21]. With increase in the prevalence of HIV, geographic distribution of subtypes has diversified to a large extent. The greatest genetic variation in HIV-1 has been found in regions where the HIV epidemic is oldest such as the regions of sub-Saharan Africa where most of the HIV-1 subtypes and many of the CRFs have been identified [22]. Kenya is one of the many countries of sub Saharan Africa region where HIV pandemic has had an overwhelming effect. Estimates suggest that by the end of the year 2005, 1.5 million people of Kenya were found to be HIV infected. In the same year, about 140,000 adults and children were estimated to have died from AIDS [23]. Epidemiological Studies have indicated that majority cases of HIV in Kenya belong to subtype A [24]. Although HIV-1 subtype A is dominant in Kenya there is an increasing prevalence of other subtypes and recombinant viruses as well. For instance CRF10 strain was first identified in western Kenya [25]. Epidemiological studies in Kenya have also reported increased prevalence in subtype C and D [26].

There is no recent published data regarding HIV subtypes in the general population of Nairobi, one of the main cities of Kenya. Few studies were carried out to track the HIV-1 subtypes in Nairobi but these studies are relatively outdated, have dealt with limited sample size [26], [27], or have targeted specific high risk groups [28]. In the present study, we investigate the prevalence of circulating HIV-1 subtypes in a sample size that is larger and more diverse than the ones studied previously.

## Materials and Methods

### Study subjects

Ethical approval for this study was obtained from the Ethical Research Council, Aga Khan University. For the study, HIV-infected residents of Nairobi were recruited at the Aga Khan Hospital, Nairobi, Kenya. Informed consent was obtained from all participants along with the data on gender, age, ethnic background, occupation, and marital status. HIV-1 status of these subjects was previously known to be positive based on their HIV-1 antibody test. All subjects who gave consent were recruited into the study, and their blood samples obtained during the year 2007.

### Extraction of genomic DNA

3–4 ml of whole blood was collected from each subject. Extraction of DNA was carried out as described previously [29]. Briefly, to 0.5 ml of peripheral blood mononuclear cells (PBMCs), 0.9 ml of 1× RBC lysing solution (0.32 M Sucrose, 1% Triton X-100, 5 mM MgCl2.6H2O, 12 mM Tris- HCl, pH 7.6) was added and centrifuged at 13,000 rpm for 1 min. After discarding the supernatant the pellet was re-extracted with 0.9 ml RBC lysing solution. After centrifugation at 13,000 rpm, the pellet was washed with 1 ml water. To the pellet, 20 µl of 20% SDS, 80 µl Proteinase K buffer (0.375 M NaCl, 0.12 M EDTA, pH 8.0) and 40 µl of 10 mg/ml Proteinase K was added to the solution which was then incubated at 56°C for 1 hour. Subsequently, 200 µl of 6 M NaCl was added to the suspension and then centrifuged at 13,000 rpm for 5 min. Supernatant was then transferred to a fresh tube and added with 400 µl of isopropanol. DNA was then pelleted by centrifugation at 13,000 rpm for 5 min. The DNA pellet was washed with 70% ethanol, air-dried, re-suspended in 100 µl of water, and stored at −20°C.

### β-globin PCR

To ascertain the quantity and quality of the extracted DNA, β-globin PCR was carried out using the previously described [30] primers PC03 (5′-ACACAACTGTGTTCACTAGC-3′) and PC04 (5′-CAACTTCATCCACGTTCACC-3′). The final 25 µl PCR mixture contained 5 µl samples, 1× PCR buffer (5× Green GoTaq® Flexi Buffer, pH 8.5), 1 mM MgCl_2,_ 200 µM dNTPs, 0.2 pmol of each primer and 0.2 U of *Taq* polymerase. Thermocycle was: denaturation at 94°C for 5 min, followed by 40 cycles of denaturation at 94°C for 30 sec, annealing at 51°C for 30 sec and extension at 72°C for 30 sec, with a final extension of at 72°C for 5 min.

### Nested PCR for *Gag* Gene

PCR amplification of complete *gag* of HIV-1 was performed with two sets of primers in a two-step nested PCR strategy. The primers used in the first round of PCR were GOPF (5′ CTCTCGACGCAGGACTCGGCTTGC-3′, nt 683–706, HXB2) and GOPR (5′- CCAATTCCCCCTATCATTTTTGG-3′, nt 2382–2404). For the second round of amplification, primers GIPF (5′- GAGGCTAGAAGGAGAGAGATGGG-3′, nt 772–794, HXB2) and GIPR (5′-TTATTGTGACGAGGGGTCGTTGCC-3′, nt 2269–2292) were used.

The reaction mixture of 25 µl for both first and second round PCR contained 1× PCR buffer (5× Green GoTaq® Flexi Buffer, pH 8.5), 2 mM MgCl_2_, 400 µM dNTPs and 0.3 U of *Taq* Polymerase. The first round of PCR was performed with 0.48 pmol of primers GOPF and GOPR. Thermocycle was: denaturation at 95°C for 5 min, followed by 35 cycles of denaturation at 95°C for 1 min, annealing at 58°C for 1 min and extension at 72°C for 1 min, with a final extension of at 72°C for 15 min.

1 µl of the first-round PCR product along with 0.48 pmol of the primers GIPF and GIPR was used for the second-round PCR. Thermocycle was: denaturation at 95°C for 5 min, followed by 35 cycles of denaturation at 95°C for 1 min, annealing at 60°C for 1 min and extension at 72°C for 1 min, with a final extension of at 72°C for 15 min. The amplified products were electrophoresed on 1.2% agarose gel, stained by ethidium bromide and visualized under ultraviolet light.

### Sequencing and Phylogenetic Analysis

Nested PCR products of *gag* gene were partially sequenced from Macrogen Inc, Korea, using the primer GSP1 (5′- CCATCAATGAGGAAGCTGC-3′, nt 1400–1418, HXB2). For subtyping and further analysis, the nucleotide sequence spanning the p24 and p7 region of *gag* gene, nt 1577–2040, HXB2 [31] (comprising 460–470 bp), was aligned with sequences from the Los Alamos HIV sequence database. This was accomplished by using the HIV BLAST Search (http://www.hiv.lanl.gov/). The samples were assigned subtypes based on the closest homology found with the subtype references in the Los Alamos database.

Using the same sequence, alignments were obtained by the Clustal X program (1.83) [32]. After alignment the positions where gaps occurred were stripped and minor manual adjustments were made using MacClade [33]. From these alignments, phylogenetic relationships were determined by using neighbor-joining method with the help of PAUP* [34]. Pairwise genetic distances were calculated with Kimura's two parameter method [35] *SimPlot* Version 3.5.1 was used for the analysis of recombinant subtypes[36] . In order to establish geographic relationship between our and previously reported strains, reference *gag* sequences from different countries were selected from the Los Alamos HIV sequence database and sequence alignment and phylogenetic analysis performed. All of the sequences were analyzed for G→A hypermutation using the consensus sequence for the appropriate subtype by the Los Alamos *Hypermut* Program (http://www.hiv.lanl.gov/content/sequence/HYPERMUT/hypermut.html).

## Results

### Subject Profile

Samples collected in the year 2007, from 69 HIV-infected subjects who consented to the study, were analyzed. Of these subjects, 32 (46.37%) were males and 37 (53.63%) were females (Table 1). Their ages ranged between 16 and 58 years (Table 1). Recruited subjects had varying ethnic backgrounds with majority being from Luo and Kikuyu communities followed by Kamba (Table 1). Distribution via occupation revealed a mixed picture: persons involved in business numbered 10 (14.49%), technicians 10 (14.49%), clerks 6 (8.69%) and the rest constituting others (Table 1). Distribution of risk groups was as follows: 76.81% had history of unprotected sex, 15.94% had history of blood transfusion whereas 4.34% had other risk factors (Table 1).

**Table 1:** Distribution of study subjects on the basis of gender, age, occupation, ethnicity and risk factors.

Characteristics	Subcategory	Distribution of Subjects
**Gender**	Male	32 (46.37%)
	Female	37 (53.63%)
**Age Groups**	11–20	2 (2.89%)
	21–30	4 (5.79%)
	31–40	34 (49.27%)
	41–50	23 (33.33%)
	51–60	6 (8.70%)
**Occupation**	Business	10 (14.50%)
	Technicians	10 (14.50%)
	Clerks	6 (8.69%)
	Drivers	5 (7.24%)
	House wives	5 (7.24%)
	Students and Teachers	4 (5.80%)
	Others	29 (42.03%)
**Ethnicity**	Luo	16 (23.18%)
	Kikuyu	16 (23.18%)
	Kamba	13 (18.84%)
	Luhya	8 (11.59%)
	Kalenjin	3 (4.34%)
	Unknown	4 (5.80%)
	Others	9 (13.04%)
**Risk Factors**	Unprotected Sex	53 (76.81%)
	Blood Transfusion & Unprotected Sex	11 (15.94%)
	Blood Transfusion	1 (1.44%)
	Polygamous	1 (1.44%)
	Unknown	3 (4.34%)

### Subtyping and Phylogenetic Analysis

**Figure 1:**
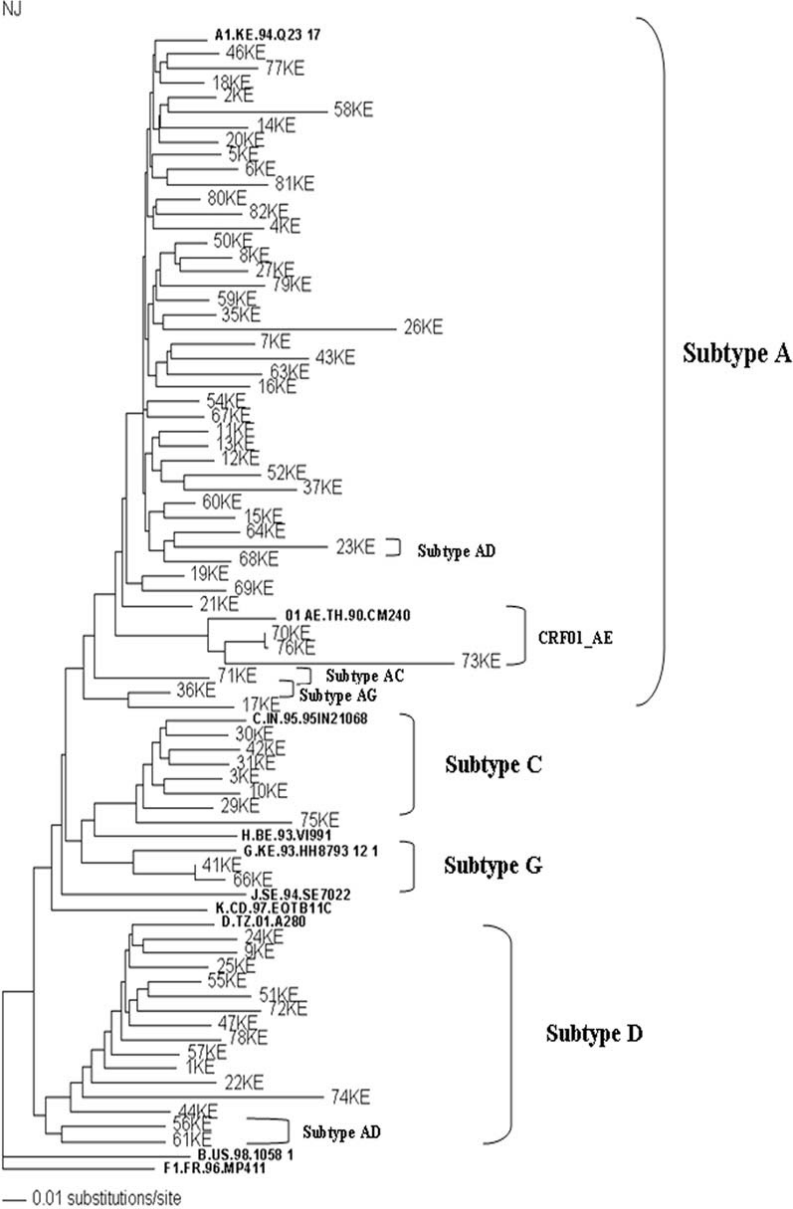
Phylogenetic analysis of HIV *gag* gene (p24-p7) sequences (nt 1577–2040, HXB2) from HIV infected Nairobi residents. This tree (neighbor-joining) was created by aligning the selected sequences with reference sequences from Los Alamos database shown in bold). The sequence F1.FR.96.MP411 was selected as the out group.

69 samples were successfully amplified for the *gag* gene in a nested PCR, followed by sequencing. Generated sequences (approximately covering 460–470 bp of p24 and p7 region of *gag* gene, nt 1577–2040, HXB2) were then used for sequence alignment using Clustal X and to construct the phylogenetic trees using neighbor-joining method with PAUP*. Alignment of sequences with reference sequences from Los Alamos database and Phylogenetic analysis revealed that 39 (56.52%) of the 69 HIV-1 subtypes were A, 13 (18.84%) were subtype D, 7 (10.14%) were subtype C and 2 (2.89%) were subtype G. *Simplot* analysis revealed a few recombinant types in our study samples: 3 (4.34%) being AD, 1 (1.44%) AC, 1 (1.44%) AG and 3 (4.34%) CRF01_AE (Table 2, Fig. 1).

**Table 2:** Distribution of HIV-1 subtypes in the studied population.

Subtypes	Number of Subjects
A	39 (56.52%)
D	13 (18.84%)
C	7 (10.14%)
G	2 (2.89%)
AD	3 (4.34%)
AC	1 (1.44%)
AG	1 (1.44%)
CRF01_AE	3 (4.34%)

### Phylogenetic Analysis for Geographic Relationship

**Figure 2:**
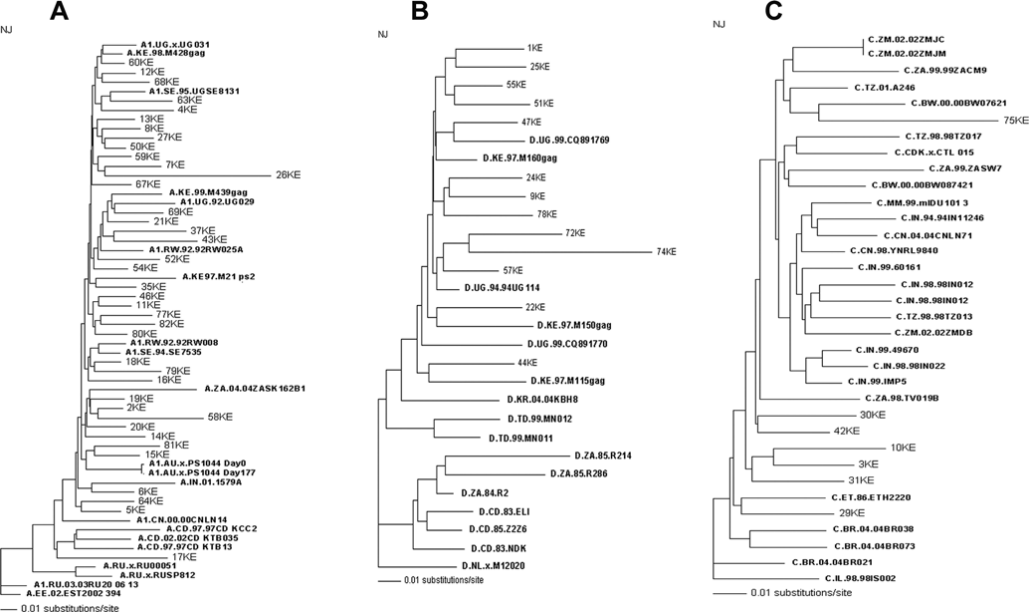
Neighbor-Joining Phylogenetic trees of the three most represented subtypes, A (Fig. 2A), D (Fig. 2B) and C (Fig. 2C), depicting geographical associations of the studied sample group with other global regions. Reference Sequences from Los Alamaos Database have been indicated in bold. Out groups selected for Fig. 2A, B, and C were, respectively, A.EE.02.EST2002 394, NL.x.M12020, and IL.98.98IS002.

For the three most represented subtypes in our study, namely, A, D and C, deeper phylogenetic analysis was performed to explore their geographic origin (Fig. 2A, B, C). Our sample subtype A sequences clustered with sequences from varied geographical regions represented by Uganda, Kenya, Sweden, Rwanda, South Africa, Australia, India, China and Democratic Republic of the Congo (Fig. 2A). These results indicate a significant diversity among our subtype A sequences, possibly indicating an older HIV-1 infection in the Nairobi population. Conversely, Phylogenetic analysis of subtype D sequences revealed a relatively lesser degree of diversity (Fig. 2B). Sequences of 10 out of 12 strains for subtype D clustered together, and with a reference sequence from Uganda indicating a strong phylogenetic relationship and lesser genetic divergence compared to subtype A sequences. Two out of twelve subtype D sequences clustered independently with reference sequences from Kenya. Lastly, subtype C sequences, in a manner similar to our subtype D sequences, clutered closely together, and with African countries (Fig. 2C). Four out of seven subtype C sequences clustered closely with reference sequence from Ethiopia while three sequences clustered with references from South Africa and Botswana. One of the sequence, 26KE, representing subtype A, was found to be hypermutated by the *Hypermut* Program at Los Alamos Database (http://www.hiv.lanl.gov/content/sequence/HYPERMUT/hypermut.html).

## Discussion

In the present study we document the genetic diversity of HIV-1 strains in the general population of Nairobi, Kenya, by sequence subtyping and phylogenetic analysis of *gag* region.

### Subtype Distribution Profile

HIV-1 subtype A was found to be the predominant type circulating in the Nairobi population followed by subtypes D, C and G. These findings suggest a consistent and continual spread of subtype A in Nairobi (Table 2). The data presented here illustrate a heterogeneous epidemic which is consistent with the previous epidemiological studies in Kenya [24], [27], [37]–[41]. Homology of Kenyan subtype A samples with multiple countries indicates that the origin of subtype A infection in these countries may be from Kenya.

We have also identified recombinants AD, AC, AG and CRF01_AE, which carry recombined subtype A genomic sequences. Occurrence of these mixed subtypes and CRFs indicates that subtype A is not only predominantly in circulation in the Kenyan population but with time, it is slowly evolving and diverging into new HIV strains. These recombinant forms might have developed from recombination between parental subtype strains within dually infected patients or through transmission of already recombined strains to the subjects studied. With any of the above possibilities it is evident that dual infections are common in this population.

### HIV-1 subtype distribution according to gender and age

Previous reports have suggested that HIV in Kenya is more prevalent in women than in men [23], in our study as well, of the recruited HIV seropositive subjects, majority was of females (37 women and 32 men). In female subjects the prevalent HIV-1 subtype was A (48.64%). The prevalence of subtype D (21.62%) and C (13.51%) in women was found to be higher as compared to men, which were, respectively, 15.62% and 6.25%.

Analysis of the patient profiles revealed that most of the study subjects belonged to age group 31–50 years. Moreover, phylogenetic analysis of the samples from these age groups indicated the prevalence of subtype G, intersubtype AD, AC and AG within the age group of 31–40, whereas the prevalence of CRF01_AE was found to be higher in the age group of 41–50 (66.67%) (Table 1).

### HIV-1 subtype distribution according to Ethnicity

In our HIV seropositive study group, Luo and Kikuyu, comprising 16 subjects each, were the most represented groups, followed by Kamba (13 subjects) (Table 1). In the Luo group, subtype A, D and its intersubtype AD were discovered while subtype C was found to be surprisingly absent. Interestingly, all subtypes discovered in this study were represented in the Kikuyu group. These ethnic groups exhibit distinct cultural behavior and practices which may play a crucial role in the transmission of HIV within and from these groups. Distribution of various ethnicities in Kenya is in the order: Kikuyu (22%), Luhya (14%), Luo (13%), Kalenjin (12%), Kamba (11%). In our HIV positive subjects, however, the order of predominance was: Luo/Kikuyu, Kamba, Luhya, and Kalenjin. A high occurrence of HIV in Luo and Kikuyu groups may have a connection with cultural and social practices. Traditional male circumcision, which in certain trials has been shown to be protective against HIV[42], is, for instance, not practiced in the Luo ethnicity [43]. Polygamy and wife inheritance, on the other hand, is common in the Luo community [44], which may also play a role in increased transmission of HIV and confinement of specific subtypes within this group. Finally, CRF01_AE was found to be more prevalent in the ethnic group Kamba (66.67%) compared to any other ethnic group. This was an interesting observation since prevalence of CR01_AE, previously reported in Central African Republic countries, Chad, Congo, and Egypt, has not been noted in the East African region (www.hiv.lanl.gov). Kamba ethnicity is distributed in the east-central and coastal Kenya. Prevalence of CR01_AE in this tribe may be attributed to their interaction with countries of Central African Republic through trade and travel.

### Phylogenetic relationship of Kenya subtypes with other countries

Phylogenetically, most of the HIV-1 subtypes in our study have shown their closest homology and association with African countries such as Kenya, Uganda, South Africa, Ethiopia, Rwanda, Botswana and Democratic Republic of Congo. These geographic associations suggest that the bulk of HIV-1 infection in Nairobi is likely to be a result of direct transmission of the virus within these countries possibly via travelers across borders. Certain viral strains bearing subtype A demonstrated closer homology to strains from India, Australia and Sweden which may indicate spread of these strains from or to Kenya. Closer relationship of our subtype D and C, but not A, sequences with African subtypes may indicate a propensity of transmission of these subtypes for specific genetic backdrops.

### Conclusion

Among the sub-Saharan African countries, Kenya has one of the highest prevalence rates of HIV-1 infection [45]. HIV infection among adults in urban areas, such as Nairobi, is almost twice (10%) as high as in rural areas (5–6%). Studies previously conducted in Nairobi have targeted particular high-risk groups [28] and/or have relied on a limited number of samples [46]. Moreover, most of these studies date a few years back, necessitating an update on general as well as molecular epidemiology data [28], [46]. Increasing variation among HIV subtypes may have implications on HIV prevention and treatment programmes. At this stage few vaccine trials in Kenya are underway or are presently in the planning stage [47], [48] and there is a need to monitor the current extent of HIV-1 subtype divergence in the infected population in different parts of the country.

## References

[1] Ho DD (1995). HIV-1 dynamics in vivo.. J Biol Regul Homeost Agents.

[2] Roberts JD, Bebenek K, Kunkel TA (1988). The accuracy of reverse transcriptase from HIV-1.. Science.

[3] Spira S, Wainberg MA, Loemba H, Turner D, Brenner BG (2003). Impact of clade diversity on HIV-1 virulence, antiretroviral drug sensitivity and drug resistance.. J Antimicrob Chemother.

[4] Tatt ID, Barlow KL, Nicoll A, Clewley JP (2001). The public health significance of HIV-1 subtypes.. Aids.

[5] Temin HM (1993). A proposal for a new approach to a preventive vaccine against human immunodeficiency virus type 1.. Proc Natl Acad Sci U S A.

[6] Gao F, Yue L, Robertson DL, Hill SC, Hui H (1994). Genetic diversity of human immunodeficiency virus type 2: evidence for distinct sequence subtypes with differences in virus biology.. J Virol.

[7] Gao F, Yue L, White AT, Pappas PG, Barchue J (1992). Human infection by genetically diverse SIVSM-related HIV-2 in west Africa.. Nature.

[8] Hahn BH, Shaw GM, De Cock KM, Sharp PM (2000). AIDS as a zoonosis: scientific and public health implications.. Science.

[9] Osmanov S, Pattou C, Walker N, Schwardlander B, Esparza J (2002). Estimated global distribution and regional spread of HIV-1 genetic subtypes in the year 2000.. J Acquir Immune Defic Syndr.

[10] Sharp PM, Robertson DL, Hahn BH (1995). Cross-species transmission and recombination of ‘AIDS’ viruses.. Philos Trans R Soc Lond B Biol Sci.

[11] Thomson MM, Perez-Alvarez L, Najera R (2002). Molecular epidemiology of HIV-1 genetic forms and its significance for vaccine development and therapy.. Lancet Infect Dis.

[12] Malim MH, Emerman M (2001). HIV-1 sequence variation: drift, shift, and attenuation.. Cell.

[13] Peeters M, Sharp PM (2000). Genetic diversity of HIV-1: the moving target.. Aids.

[14] Robertson DL, Anderson JP, Bradac JA, Carr JK, Foley B (2000). HIV-1 nomenclature proposal.. Science.

[15] Tee KK, Li XJ, Nohtomi K, Ng KP, Kamarulzaman A (2006). Identification of a novel circulating recombinant form (CRF33_01B) disseminating widely among various risk populations in Kuala Lumpur, Malaysia.. J Acquir Immune Defic Syndr.

[16] Essex M (1999). Human immunodeficiency viruses in the developing world.. Adv Virus Res.

[17] Kuiken C, Thakallapalli R, Esklid A, de Ronde A (2000). Genetic analysis reveals epidemiologic patterns in the spread of human immunodeficiency virus.. Am J Epidemiol.

[18] Kandathil AJ, Ramalingam S, Kannangai R, David S, Sridharan G (2005). Molecular epidemiology of HIV.. Indian J Med Res.

[19] Holmes EC (2001). On the origin and evolution of the human immunodeficiency virus (HIV).. Biol Rev Camb Philos Soc.

[20] Brennan CA, Bodelle P, Coffey R, Harris B, Holzmayer V (2006). HIV global surveillance: foundation for retroviral discovery and assay development.. J Med Virol.

[21] Kato S, Saito R, Hiraishi Y, Kitamura N, Matsumoto T (2003). Differential prevalence of HIV type 1 subtype B and CRF01_AE among different sexual transmission groups in Tokyo, Japan, as revealed by subtype-specific PCR.. AIDS Res Hum Retroviruses.

[22] McCutchan FE (2000). Understanding the genetic diversity of HIV-1.. Aids.

[23] UNAIDS/WHO (2006). 2006 Report on the global AIDS epidemic.

[24] Dowling WE, Kim B, Mason CJ, Wasunna KM, Alam U (2002). Forty-one near full-length HIV-1 sequences from Kenya reveal an epidemic of subtype A and A-containing recombinants.. Aids.

[25] Songok EM, Lihana RW, Kiptoo MK, Genga IO, Kibaya R (2003). Identification of env CRF-10 among HIV variants circulating in rural western Kenya.. AIDS Res Hum Retroviruses.

[26] Robbins KE, Kostrikis LG, Brown TM, Anzala O, Shin S (1999). Genetic analysis of human immunodeficiency virus type 1 strains in Kenya: a comparison using phylogenetic analysis and a combinatorial melting assay.. AIDS Res Hum Retroviruses.

[27] Poss M, Gosink J, Thomas E, Kreiss JK, Ndinya-Achola J (1997). Phylogenetic evaluation of Kenyan HIV type 1 isolates.. AIDS Res Hum Retroviruses.

[28] Lihana RW, Khamadi SA, Kiptoo MK, Kinyua JG, Lagat N (2006). HIV type 1 subtypes among STI patients in Nairobi: a genotypic study based on partial pol gene sequencing.. AIDS Res Hum Retroviruses.

[29] Khan S, Rai MA, Khanani MR, Khan MN, Ali SH (2006). HIV-1 subtype A infection in a community of intravenous drug users in Pakistan.. BMC Infect Dis.

[30] Saiki RK, Gelfand DH, Stoffel S, Scharf SJ, Higuchi R (1988). Primer-directed enzymatic amplification of DNA with a thermostable DNA polymerase.. Science.

[31] Heyndrickx L, Janssens W, Zekeng L, Musonda R, Anagonou S (2000). Simplified strategy for detection of recombinant human immunodeficiency virus type 1 group M isolates by gag/env heteroduplex mobility assay. Study Group on Heterogeneity of HIV Epidemics in African Cities.. J Virol.

[32] Jeanmougin F, Thompson JD, Gouy M, Higgins DG, Gibson TJ (1998). Multiple sequence alignment with Clustal X.. Trends Biochem Sci.

[33] Maddison DR, Maddison WP (2000). MacClade version 4 Analysis of phylogeny and character evolution.

[34] Swofford DL (2001). PAUP*: Phylogenetic analysis using parsimony (*and other methods) beta version 4.0b8.

[35] Kimura M (1980). A simple method for estimating evolutionary rates of base substitutions through comparative studies of nucleotide sequences.. J Mol Evol.

[36] Lole KS, Bollinger RC, Paranjape RS, Gadkari D, Kulkarni SS (1999). Full-length human immunodeficiency virus type 1 genomes from subtype C-infected seroconverters in India, with evidence of intersubtype recombination.. J Virol.

[37] Janssens W, Heyndrickx L, Fransen K, Temmerman M, Leonaers A (1994). Genetic variability of HIV type 1 in Kenya.. AIDS Res Hum Retroviruses.

[38] Morison L, Buve A, Zekeng L, Heyndrickx L, Anagonou S (2001). HIV-1 subtypes and the HIV epidemics in four cities in sub-Saharan Africa.. Aids.

[39] Neilson JR, John GC, Carr JK, Lewis P, Kreiss JK (1999). Subtypes of human immunodeficiency virus type 1 and disease stage among women in Nairobi, Kenya.. J Virol.

[40] Zachar V, Goustin AS, Zacharova V, Hager H, Koppelhus U (1996). Genetic polymorphism of envelope V3 region of HIV type 1 subtypes A, C, and D from Nairobi, Kenya.. AIDS Res Hum Retroviruses.

[41] Yang C, Li M, Newman RD, Shi YP, Ayisi J (2003). Genetic diversity of HIV-1 in western Kenya: subtype-specific differences in mother-to-child transmission.. Aids.

[42] Bailey RC, Moses S, Parker CB, Agot K, Maclean I (2007). Male circumcision for HIV prevention in young men in Kisumu, Kenya: a randomised controlled trial.. Lancet.

[43] Auvert B, Buve A, Lagarde E, Kahindo M, Chege J (2001). Male circumcision and HIV infection in four cities in sub-Saharan Africa.. Aids.

[44] Luginaah I, Elkins D, Maticka-Tyndale E, Landry T, Mathui M (2005). Challenges of a pandemic: HIV/AIDS-related problems affecting Kenyan widows.. Soc Sci Med.

[45] Mertens TE, Belsey E, Stoneburner RL, Beer DL, Sato P (1995). Global estimates and epidemiology of HIV-1 infections and AIDS: further heterogeneity in spread and impact.. Aids.

[46] Belda FJ, Mwchari C, Hawken M, Barlow KL, Clewley JP (1997). HIV-1 subtypes in Nairobi, Kenya.. J Acquir Immune Defic Syndr Hum Retrovirol.

[47] Hanke T, McMichael AJ (2000). Design and construction of an experimental HIV-1 vaccine for a year-2000 clinical trial in Kenya.. Nat Med.

[48] Mwau M, Cebere I, Sutton J, Chikoti P, Winstone N (2004). A human immunodeficiency virus 1 (HIV-1) clade A vaccine in clinical trials: stimulation of HIV-specific T-cell responses by DNA and recombinant modified vaccinia virus Ankara (MVA) vaccines in humans.. J Gen Virol.

